# Statistical Analysis of Multiple Vaccine Effectiveness Against COVID-19 Variants: Integrating Immune Response Dynamics

**DOI:** 10.7759/cureus.76705

**Published:** 2024-12-31

**Authors:** Jiwoo Kim, Hyosoon Jung

**Affiliations:** 1 Medicine, John A. Burns School of Medicine, University of Hawaiʻi at Mānoa, Honolulu, USA; 2 Preventive Medicine, Defense Health Agency, Area IV, Daegu, KOR

**Keywords:** covid-19, delta variants, immune response, multiple doses, multiple linear regression, omicron variants, vaccine

## Abstract

The study aims to evaluate the association between multiple COVID-19 vaccine doses and daily confirmed cases, incorporating immune response delays to better understand vaccination efficacy. We investigated the effectiveness of multiple COVID-19 vaccinations, with particular emphasis on viral variants, by integrating immune response dynamics into the statistical analysis to provide a more accurate assessment of vaccination outcomes. Detailed data on vaccination numbers and confirmed COVID-19 cases were obtained from the official Korean Ministry of the Interior and Safety website. Multiple linear regression was applied to evaluate the effectiveness of each vaccine dose against different COVID-19 variants. This approach offered a clearer understanding of how vaccination influenced the spread of COVID-19 over time. By accounting for delay periods associated with immune responses, we aimed to provide more accurate insights into the effectiveness of each vaccine dose. The results underscored the impact of multiple vaccinations when incorporating immune response timing and revealed notable differences in the responses of the Delta and Omicron variants to multiple doses. While significant effectiveness was observed against the Omicron variant, a similar positive effect was not evident for the Delta variant.

## Introduction

The COVID-19 outbreak, which began in Wuhan, China, in December 2019, escalated rapidly into a global crisis, leading the World Health Organization (WHO) to declare it a pandemic by March 2020 [1,2]. The unprecedented spread of the virus strained healthcare systems worldwide, highlighting the urgent need for effective vaccines. In response, researchers and pharmaceutical companies collaborated to develop and distribute several vaccines, including Pfizer-BioNTech’s BNT162b2, Moderna’s mRNA-1273, AstraZeneca’s AZD1222, and Novavax’s NVX-CoV2373 [3-6]. Vaccination campaigns began in December 2020, with the goals of achieving herd immunity, protecting high-risk populations, and reducing the burden on healthcare systems [7,8]. Despite the promising results of these vaccines against the original strain of SARS-CoV-2, their efficacy against emerging variants, such as Delta and Omicron, raised significant questions.

This study addresses the evolving challenge posed by viral mutations by analyzing the effectiveness of multiple vaccine doses against COVID-19 variants, focusing on data from Seoul, South Korea. Seoul’s high vaccination coverage, including both initial and booster doses, made it an ideal setting for this analysis [9,10]. Using multiple linear regression analysis, we evaluated the influence of 1-dose, 2-dose, and 3-dose regimens on daily confirmed COVID-19 cases across different viral variants. A key feature of this study is the introduction of a delay period into the regression model to account for the time required for an immune response to develop after vaccination. This approach allowed for a more precise evaluation of vaccine effectiveness over time. Our analysis incorporated statistical measures, including p-values to assess the significance of vaccine effectiveness and adjusted R-squared values to quantify the contributions of each dose to changes in confirmed case numbers. The findings provide critical insights into the dynamic relationship between vaccination efforts and the spread of COVID-19 variants. By accounting for immune response timing, this study offers a comprehensive understanding of how different vaccine regimens impacted daily case trends and highlights the varying effectiveness of these doses against the Delta and Omicron variants.

Understanding the dynamics of COVID-19 vaccination and its impact on daily confirmed case counts is critical for informing public health strategies. This study seeks to evaluate the statistical relationship between the number of vaccine doses administered and daily confirmed cases of COVID-19, with a particular focus on incorporating the delays associated with immune response development. By integrating these delays into the analysis, we aim to provide a more nuanced understanding of vaccine efficacy over time.

## Materials and methods

Data collection and integration

To assess the impact of multiple COVID-19 vaccine doses on viral variants while accounting for delay periods in daily confirmed cases, statistical regression analyses were performed [11]. The study used two datasets for our analysis: "Confirmed Cases of COVID-19 in Seoul City" and "COVID-19 Vaccination Status in Seoul City." These datasets were sourced from the official website of the Korean Ministry of the Interior and Safety [12]. Confirmed cases were determined through positive polymerase chain reaction (PCR) test results detecting SARS-CoV-2 RNA. Sample entries from this dataset are displayed in Table 1. For the analysis, multiple linear regression was applied, with adjustments made to the last column (confirmed cases) to reflect the effects of the delay period, ensuring a more accurate evaluation of vaccine effectiveness.

**Table 1:** Daily numbers of vaccinated individuals with multiple doses and confirmed COVID-19 cases among the vaccine-eligible population from March 12, 2022, to March 21, 2022, covering 10 days of data as an example.

Date	Vaccine-eligible population	First dose	Second dose	Third dose	Confirmed cases
12-Mar-22	9401888	497	663	6639	140
13-Mar-22	9401888	1	5	138	112
14-Mar-22	9401888	921	1104	9062	112
15-Mar-22	9401888	431	645	6226	79
16-Mar-22	9401888	313	286	5623	120
17-Mar-22	9401888	518	585	8713	124
18-Mar-22	9401888	938	1048	13986	146
19-Mar-22	9401888	380	369	5707	120
20-Mar-22	9401888	9	7	112	125
21-Mar-22	9401888	803	757	9000	104

Study design

This study used an ecological approach, focusing on the vaccine-eligible population of Seoul, which comprised 9,401,888 people as of March 12, 2022. This approach was chosen to examine how vaccine doses influenced daily confirmed COVID-19 cases at the metropolitan city level. As of November 17, 2024, four types of vaccines had been administered in South Korea, including Seoul: Pfizer-BioNTech (46.10%), Oxford-AstraZeneca (45.22%), Moderna (4.62%), and Janssen (Johnson & Johnson) (4.06%) [13]. By analyzing the relationship between these vaccines, their doses, and COVID-19 case trends, the study provides valuable insights for public health strategies. This research focused on how multiple vaccine doses impacted COVID-19 variants by examining daily confirmed cases while accounting for a delay period between vaccination and case reporting. In our earlier work, we applied multiple linear regression to assess the effects of multiple vaccinations on daily confirmed COVID-19 cases without considering delay periods [14]. In this study, we introduce a method that incorporates the timing of the immune response, offering more accurate results compared to approaches that overlook delay periods.

To evaluate the impact of multiple vaccinations on the Delta and Omicron variants, multiple linear regression analyses were performed and the most suitable regression equation was identified based on p-values and adjusted R-squared values, with serial delay periods tested for optimization.

Study period

This study analyzed data from April 21, 2021, to March 14, 2022, to capture the progress of COVID-19 vaccination campaigns in Seoul. Although vaccinations in South Korea, including Seoul, began on February 26, 2021, detailed data on vaccination numbers and daily confirmed cases only became available starting April 21, 2021. Second doses were administered beginning in mid-March 2021, approximately three weeks after the first doses, enabling the analysis of both doses starting from April 21, 2021. Data collection for the third dose commenced on October 13, 2021, and for the fourth dose on March 15, 2022. During this period, the Delta and Omicron variants were the dominant COVID-19 strains. The Delta variant was first detected in South Korea in May 2021, with its rapid spread coinciding with the rollout of second doses. The Omicron variant began spreading in December 2021 and surged in early 2022, overlapping with the administration of third doses. This timeline provided a unique opportunity to study the relationship between vaccination efforts and the prevalence of these variants.

Statistical analysis

To evaluate the impact of each vaccine dose on daily confirmed COVID-19 cases while accounting for a delay period, multiple linear regression analysis was employed [11]. This analytical approach facilitated the modeling of the relationship between vaccination doses and daily confirmed cases, where daily confirmed cases served as the dependent variable and vaccination doses were treated as independent variables. By incorporating immune response delays, we aimed to more accurately capture the temporal dynamics of vaccine efficacy in reducing case numbers. Although all variables were discrete, multiple linear regression was deemed appropriate due to the nearly linear relationship observed between vaccination counts and daily confirmed cases. This suitability was further supported by the normality and homoscedasticity of residuals. While Poisson regression is typically preferred for count data, it was not applied due to significant overdispersion in the daily confirmed cases data [11]. Additionally, negative binomial regression was unsuitable because of multicollinearity between the first and second doses, arising from their similar daily vaccination patterns. This similarity occurred because the two doses were administered within a three-week interval to a comparable number of individuals.

In this analysis, a delay period was introduced between the independent variables (cumulative vaccination counts) and the dependent variable (daily confirmed cases) to measure vaccine effectiveness while accounting for the time required for an immune response to develop. By identifying the optimal regression lag within this delay period, the study aimed to provide a more accurate assessment of vaccine effectiveness in relation to immune response dynamics. The inclusion of delay periods in the regression model represents a novel approach to account for the immune response timeline following vaccination. Specifically, our study integrates biologically informed lag periods directly into the regression framework. This allows for more accurate attribution of observed changes in daily COVID-19 case numbers to the vaccination effect, preventing potential temporal misalignment between the cause (vaccine administration) and the effect (reduction in cases). This methodological innovation enhances the model’s ability to better reflect real-world vaccine effectiveness, providing more reliable estimates of the impact of vaccination over time. All statistical analyses, including the multiple linear regression, were conducted using Minitab statistical software (version 22; Minitab, LLC, State College, Pennsylvania, USA).

## Results

**Figure 1:**
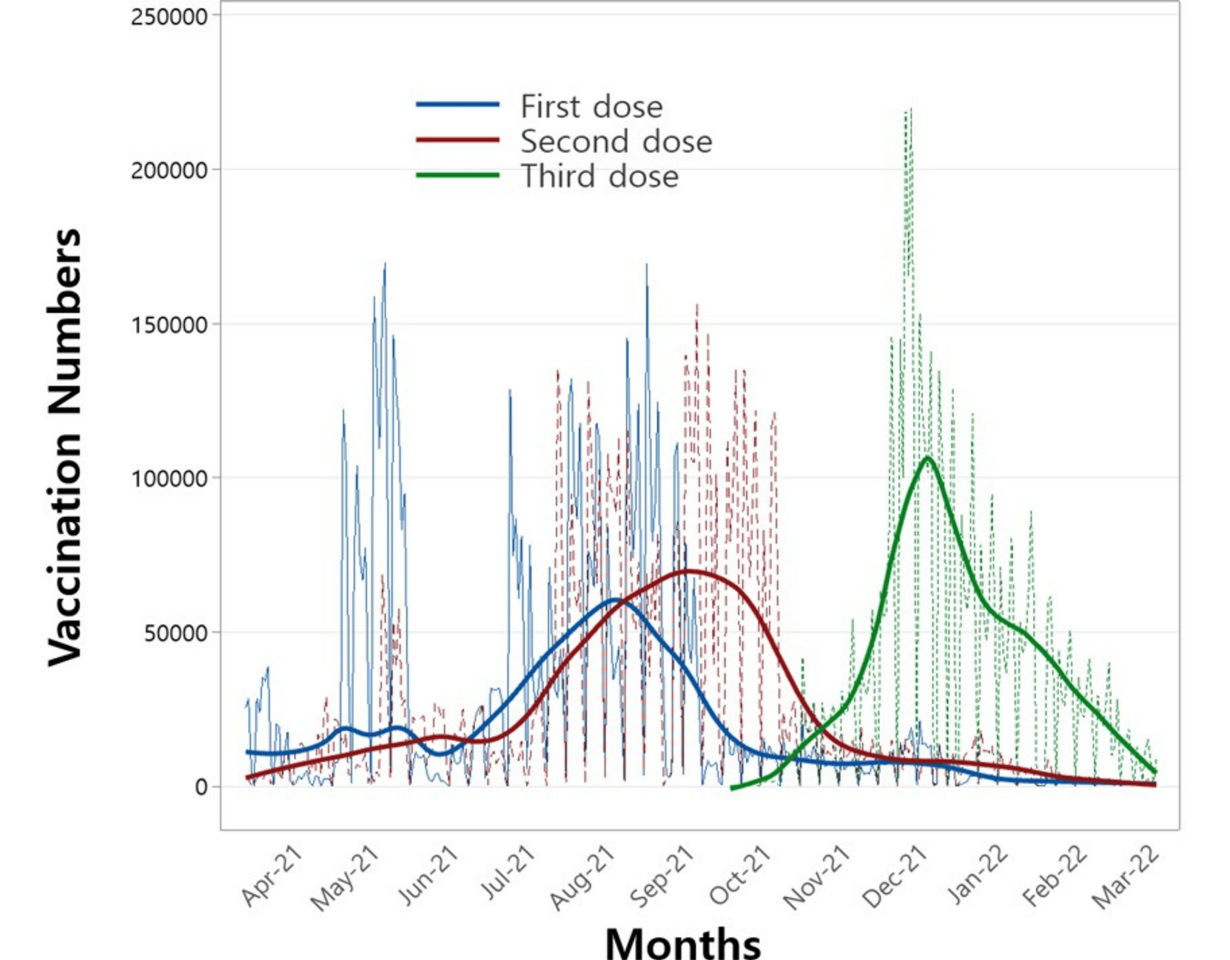
Number of vaccinations administered for the first, second, and third doses over the study period.

This study employed a statistical approach to assess the effectiveness of multiple vaccine doses against COVID-19 variants, incorporating their correlation with immune response time. Before performing the regression analysis, vaccination counts were visualized in Figure 1. The figure illustrates the daily distribution of first, second, third, and fourth doses administered during the study period, with solid lines representing smoothed trends to highlight the average patterns of vaccine administration for each dose.

**Figure 2:**
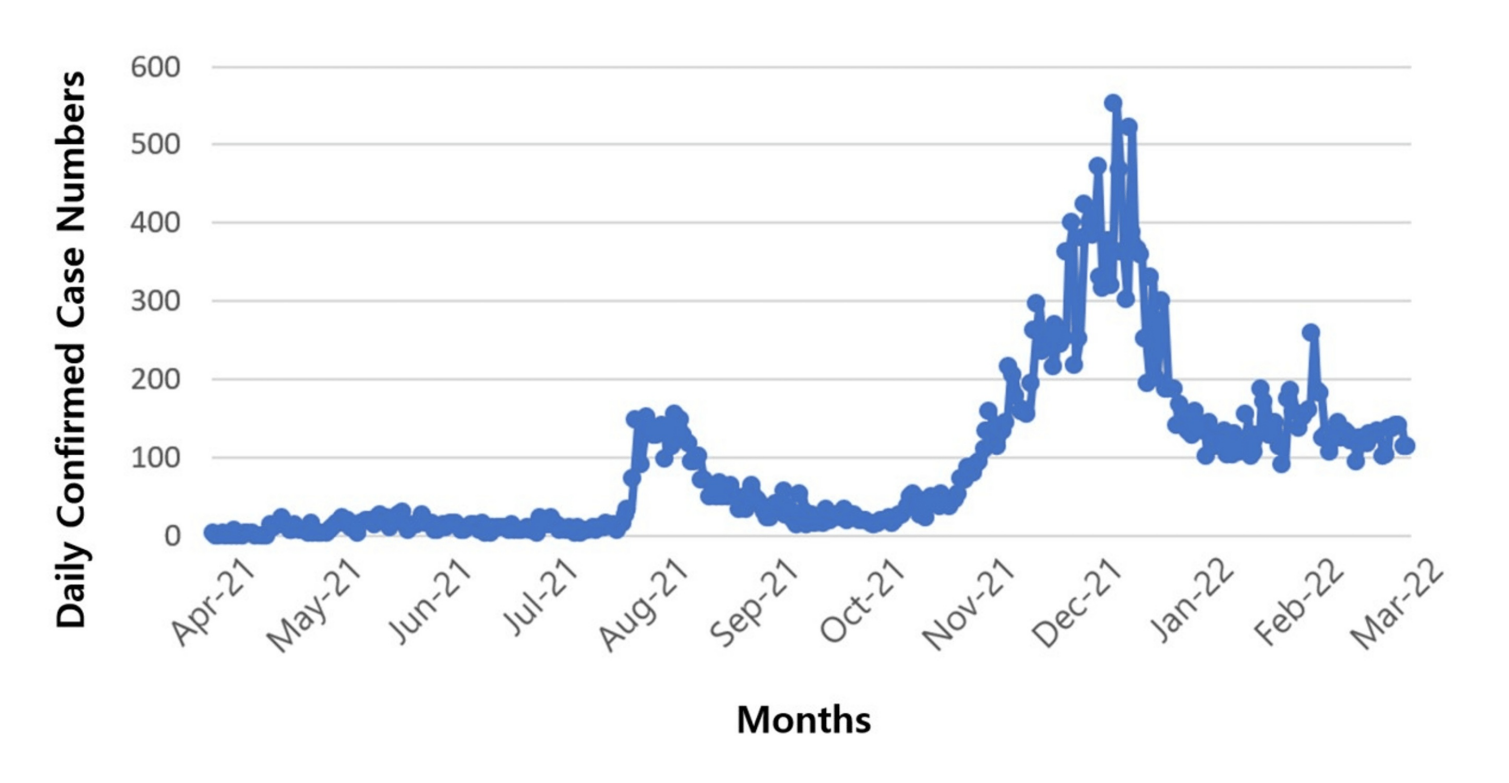
Confirmed COVID-19 cases during the administration of the first, second, and third vaccine doses over the study period.

Figure 2 depicts the daily confirmed COVID-19 cases during the study period. Two notable peaks are evident: the first, around August 2021, aligns with the emergence of the Delta variant, while the second, in December 2021, corresponds to the spread of the Omicron variant. Notably, the timing of these peaks in Figure 2 aligns closely with the peaks of the second and third vaccine doses shown in Figure 1. This alignment underscores the potential value of examining the relationship between these variants and the administration of multiple vaccine doses to derive meaningful public health insights.

Table 2 presents the results of the multiple linear regression analysis, which examines the relationship between vaccination counts and confirmed COVID-19 cases during the administration periods of the first, second, and third vaccine doses. The table includes p-values for delay periods ranging from 0 to 16 days, corresponding to the 1-dose (first dose), 2-dose (first and second doses), and 3-dose (first, second, and third doses) regimens. Notably, the 2-dose analysis was conducted for two distinct periods: one including and one excluding the Delta variant duration. The p-values from the 2-dose analysis, including the Delta variant duration, were not significant as the delay period increased, prompting an additional analysis that excluded the Delta variant duration. This suggests that the first and second vaccine doses may not offer effective protection against the Delta variant. Consequently, the Delta variant period should be excluded from further analysis of the immune response to ensure a more accurate assessment of vaccine efficacy for subsequent variants.

**Table 2:** Results of multiple linear regression analysis showing p-values for 1-dose, 2-dose, and 3-dose COVID-19 vaccine periods during the Delta and Omicron variant phases.

Delay period	1-dose	2-dose				3-dose with Omicron variant duration		
		With Delta variant duration		Without Delta variant duration				
									First dose	First dose	Second dose	First dose	Second dose	First dose	Second dose	Third dose
	0	0.019	0.000	0.003	0.001	0.004	0.000	0.000	0.000
1	0.014	0.000	0.006	0.000	0.001	0.000	0.000	0.000
2	0.009	0.000	0.009	0.000	0.000	0.000	0.000	0.000
3	0.006	0.000	0.018	0.000	0.000	0.000	0.000	0.000
4	0.004	0.000	0.038	0.000	0.000	0.000	0.000	0.000
5	0.002	0.000	0.083	0.000	0.000	0.000	0.000	0.000
6	0.001	0.001	0.152	0.000	0.000	0.000	0.000	0.000
7	0.001	0.002	0.233	0.000	0.000	0.000	0.000	0.000
8	0.001	0.005	0.418	0.000	0.000	0.000	0.000	0.000
9	0.001	0.010	0.601	0.000	0.000	0.000	0.000	0.000
10	0.001	0.019	0.826	0.000	0.000	0.000	0.000	0.000
11	0.001	0.033	0.956	0.000	0.000	0.000	0.000	0.000
12	0.000	0.064	0.667	0.000	0.000	0.000	0.000	0.000
13	0.000	0.096	0.504	0.000	0.000	0.000	0.000	0.000
14	0.000	0.101	0.482	0.000	0.000	0.000	0.000	0.000
15	0.001	0.166	0.293	0.000	0.000	0.000	0.000	0.000
16	0.001	0.212	0.207	0.000	0.000	0.000	0.000	0.000

**Figure 3:**
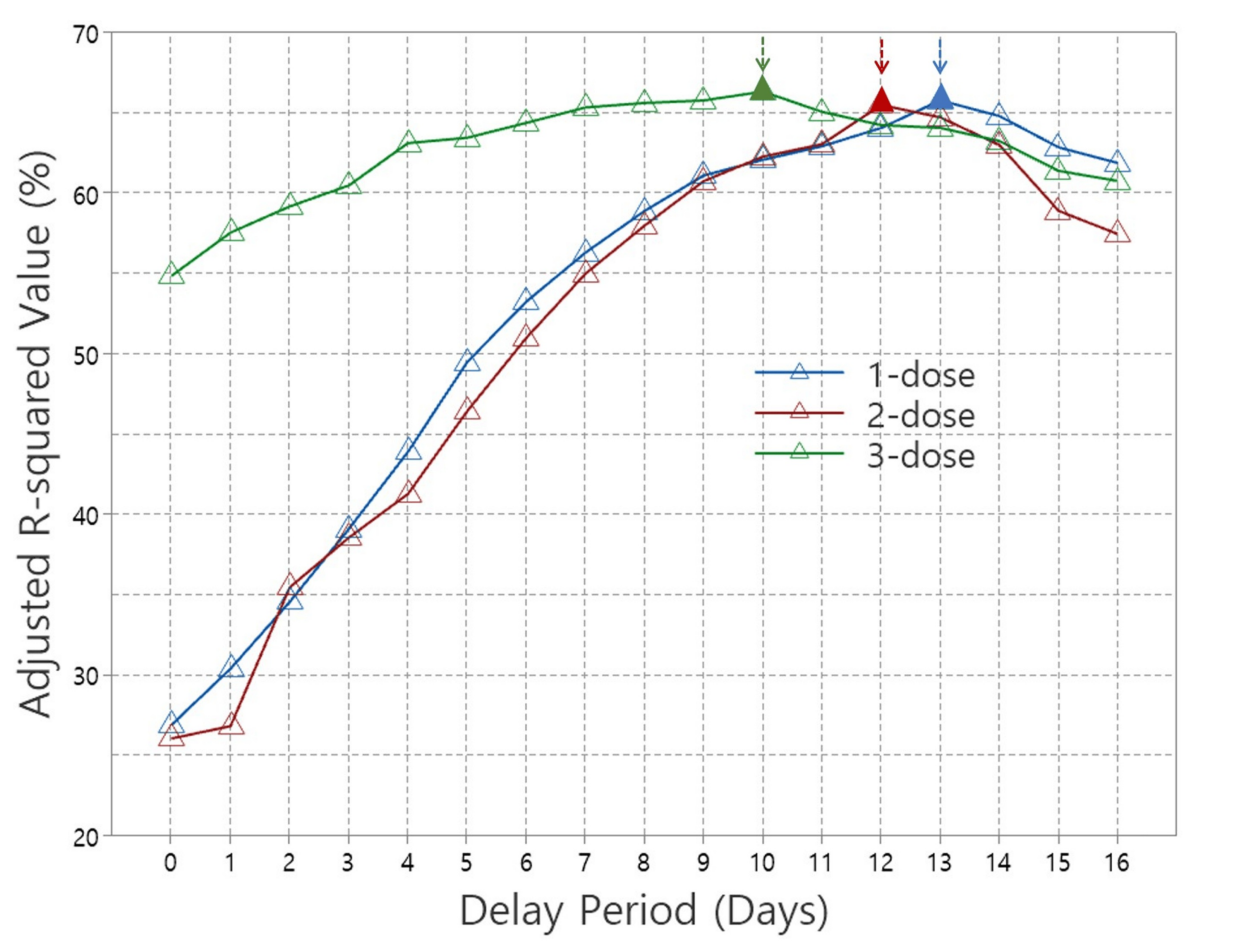
Adjusted R-squared values from multiple linear regressions for 1-dose, 2-dose, and 3-dose COVID-19 vaccines across the delay period.

Figure 3 shows the normalized adjusted R-squared values from multiple linear regressions of the 1-dose, 2-dose, and 3-dose, plotted against delay period dates. The adjusted R-squared values represent the proportion of variance in confirmed cases explained by the regression model, reflecting the contribution of each vaccine dose. Notably, all adjusted R-squared values shown in Figure 3 are statistically significant, with p-values of 0.05 or lower, as indicated in Table 2. For the 2-dose data, results excluding the Delta variant duration were used. The 4-dose data were not plotted due to non-significant p-values for most delay periods. A key observation in Figure 3 is the delay periods where the adjusted R-squared values reach their maximum, denoted by solid triangles with arrows. These peak values occur at 13 days for 1-dose, 12 days for 2-dose, and 10 days for 3-dose, suggesting that as the number of doses increases, the optimal delay period shortens.

## Discussion

It is important to note that previous studies have demonstrated positive results regarding the effectiveness of COVID-19 booster vaccines [15-19]. In particular, the third dose has been shown to reduce hospitalizations, severe illness, and COVID-19-related deaths by more than 80% [15,16]. Additionally, the fourth dose has been found to significantly reduce confirmed cases, symptomatic COVID-19 cases, hospitalizations, and deaths across all age groups [17-19]. Our previous study highlighted statistically significant effects for the first, second, and third vaccine doses; however, the fourth dose did not show a significant effect on confirmed cases [14]. In that study, we did not account for immune response time and focused solely on a delay period of 0 (i.e., same-day vaccination and confirmed cases). In contrast, the present study evaluates the effectiveness of multiple vaccine doses against COVID-19 variants that emerged during the vaccination process. This study incorporates varying delay periods to better capture immune response dynamics. By systematically varying the delay periods in the regression analyses, our goal is to identify the optimal timeframe that reflects the effectiveness of multiple vaccine doses on confirmed cases while accounting for the time required for immune responses to develop post-vaccination. The immune response time includes the activation and proliferation of immune cells, antibody production, and the establishment of immunity.

The primary COVID-19 variants of concern during the study period were the Delta and Omicron variants. COVID-19 variants are mutations of the original virus that can alter its characteristics, such as transmissibility and vaccine resistance. The emergence of variants like Delta and Omicron has raised concerns about the effectiveness of vaccines initially developed for the original strain, as their efficiency may vary depending on the specific variant. The Delta variant, known for its high transmissibility, led to breakthrough infections even among fully vaccinated individuals. Despite this, vaccinated individuals generally experienced milder symptoms compared to those who were unvaccinated [20,21]. First identified in India in late 2020, the Delta variant quickly became the dominant strain in many countries due to its enhanced ability to spread compared to earlier variants. In contrast, the Omicron variant posed even greater challenges, as its mutations allowed it to partially evade the immune response generated by vaccines. However, studies have shown that booster doses significantly enhanced protection against Omicron, improving antibody responses and reducing the risk of severe disease [17,22,23]. First identified in South Africa in late 2021, the Omicron variant spread rapidly due to its high transmissibility and a large number of mutations in the spike protein, making it more effective at evading immunity than earlier variants.

The Delta variant emerged during the administration of the first and second doses, while the Omicron variant appeared during the third-dose period. This timing highlights the evolving challenge of vaccine effectiveness against the two variants. We applied multiple linear regression to analyze p-values and adjusted R-squared values across different delay periods to assess the effectiveness of multiple vaccinations on both variants. P-values indicate the statistical significance of vaccine dose effectiveness, while adjusted R-squared values represent the degree to which each vaccine dose contributes to explaining variations in confirmed cases. We evaluated the effectiveness of 1-dose, 2-dose, and 3-dose regimens on daily confirmed cases and their impact on the Delta and Omicron variants across delay periods ranging from 0 to 16 days. The corresponding p-values are summarized in Table 2.

For the 2-dose analysis, results were presented both with and without the Delta variant duration. Including the Delta variant duration yielded mostly non-significant p-values across the delay periods, suggesting that the 2-dose vaccination was not significantly effective against the Delta variant. Interestingly, all p-values became significant when the Delta variant duration was excluded. In contrast, during the 3-dose period, which coincided with the emergence of the Omicron variant, significant p-values were observed throughout, indicating that multiple vaccinations provided sufficient protection against Omicron. These results underscore the varying effectiveness of multiple vaccination regimens against the Delta and Omicron variants. Additionally, it should be noted that the analysis for the 4-dose regimen revealed that all p-values, except for one on Day 10, were above 0.05, indicating a lack of statistical significance. This finding aligns with our previous study, which reported no significant effect of fourth doses on confirmed cases [14].

The optimal delay period for achieving the highest vaccination effectiveness is expected to decrease with each additional dose, consistent with immune response dynamics. In this study, the delay period applied in the multiple linear regression analysis aimed to identify the time point at which multiple vaccine doses exert their optimal effect. We used the adjusted R-squared value, a key regression result, to analyze this relationship across different delay periods for the 1-dose, 2-dose (excluding the Delta period), and 3-dose regimens that demonstrated statistical significance, as shown in Figure 3. In the figure, the peak adjusted R-squared values are represented by solid triangles with arrows, indicating the delay period at which each vaccination regimen achieved optimal statistical performance. This finding aligns with our expectation that the optimal immune response time decreases as the number of doses increases. The maximum adjusted R-squared values were observed at 13 days for the 1-dose regimen, 12 days for the 2-dose regimen (excluding the Delta period), and 10 days for the 3-dose regimen.

It should be mentioned that this study has several limitations. It focused on data from Seoul, South Korea, which limits the generalizability of the findings to other regions with different demographics, vaccination coverage, or public health policies. Furthermore, the use of multiple linear regression models assumes linearity and does not account for complex, nonlinear relationships between vaccination regimens and COVID-19 case trends. Additionally, factors such as waning immunity, behavioral changes, adherence to non-pharmaceutical interventions, as well as vaccination hesitancy, and compliance with public health measures, were not explicitly accounted for in the analysis. These factors may have influenced the observed vaccine effectiveness and should be considered in future studies to provide a more comprehensive understanding of vaccination outcomes. Future research should aim to incorporate individual-level data, explore nonlinear modeling approaches, and evaluate long-term vaccine effectiveness across broader contexts.

## Conclusions

This study statistically evaluated the effectiveness of multiple COVID-19 vaccine doses against variants such as Delta and Omicron, incorporating immune response dynamics into the analysis. The results revealed that while the 2-dose regimen did not demonstrate significant efficacy against the Delta variant, the 3-dose regimen, which coincided with the emergence of the Omicron variant, exhibited sustained and significant protection. This suggests that booster doses enhance immunity, particularly against Omicron variants. Additionally, the study found that the optimal delay period for vaccine effectiveness decreased with each additional dose, supporting the hypothesis that immune response time shortens as the number of doses increases. Specifically, the 1-dose regimen showed peak effectiveness at a delay period of 13 days, the 2-dose regimen at 12 days (excluding the Delta period), and the 3-dose regimen at 10 days.
